# Reconfigurable transmissive metasurface with a combination of scissor and rotation actuators for independently controlling beam scanning and polarization conversion

**DOI:** 10.1038/s41378-024-00671-y

**Published:** 2024-03-21

**Authors:** Chhunheng Lor, Ratanak Phon, Sungjoon Lim

**Affiliations:** 1 Intelligent Semiconductor Engineering, Chung-Ang University, Heukseok-Dong, Dongjak-Gu, Seoul, 06974 Republic of Korea; 2 School of Electrical and Electronic Engineering, Chung-Ang University, Heukseok-Dong, Dongjak-Gu, Seoul, 06974 Republic of Korea

**Keywords:** Electrical and electronic engineering, Nanocavities

## Abstract

Polarization conversion and beam scanning metasurfaces are commonly used to reduce polarization mismatch and direct electromagnetic waves in a specific direction to improve the strength of a wireless signal. However, identifying suitable active and mechanically reconfigurable metasurfaces for polarization conversion and beam scanning is a considerable challenge, and the reported metasurfaces have narrow scanning ranges, are expensive, and cannot be independently controlled. In this paper, we propose a reconfigurable transmissive metasurface combined with a scissor and rotation actuator for independently controlling beam scanning and polarization conversion functions. The metasurface is constructed with rotatable unit cells (UCs) that can switch the polarization state between right-handed (RHCP) and left-handed circular polarization (LHCP) by flipping the UCs to reverse their phase variation. Moreover, independent beam scanning is achieved using the scissor actuator to linearly change the distance between the UCs. Numerical and experimental results confirm that the proposed metasurface can perform beam scanning in the range of 28° for both the positive and negative regions of a radiation pattern (RHCP and LHCP beams) at an operational frequency of 10.5 GHz.

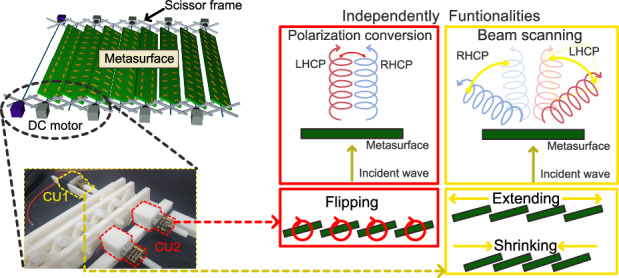

## Introduction

Reconfigurable metasurfaces are planar surfaces composed of an array of subwavelengths and can be reconfigured to engineer their light properties, such as amplitude, phase, and polarization, for electromagnetic (EM) wave manipulation. In contrast, a reconfigurable transmissive metasurface manipulates the transmission wave toward the incident wave direction. Recent reports on reconfigurable metasurfaces present two important functions: polarization conversion and beam scanning. Polarization conversion is a technique used to convert the polarization state of an EM wave, and beam scanning refers to the ability to continuously steer an incident EM wave in a desired direction. These two functions can be used to enhance image sensing^1–5^, high-resolution imaging^6–10^, and radar systems^11–15^ and improve communication efficiency^16–28^ in cases of multiple polarization states and non‒line-of-sight wave propagation.

A reconfigurable metasurface conventionally controls the functionalities of polarization conversion and beam scanning by electrically, thermal dynamically, or mechanically adjusting the phase and magnitude of a radiofrequency (RF) element array with phase and spacing modulation techniques^29–31^. A phase-modulation technique is used to configure the wave-shifting function of the RF elements by changing their EM conductivity or physical geometry. Conversely, spacing modulation techniques are employed to configure the space between the elements in an array to manipulate the EM wave direction by altering the phase distribution to steer the beam angle. Numerous integration designs incorporating both polarization conversion and beam scanning metasurfaces have been proposed with remarkable capabilities in controlling polarization states and beam direction angles. Impressively, these metasurfaces have demonstrated the potential for control^32–47^, even indicating the intriguing possibility of human-mind-based manipulation^48,49^. However, the integration of both functionalities is still limited by the ability of the metasurface to switch the polarization states, narrow scanning ranges, frequency dependence, and the inability to control the functions independently. Designing a metasurface for transmissive wave manipulation is an additional challenge.

**Fig. 1:**
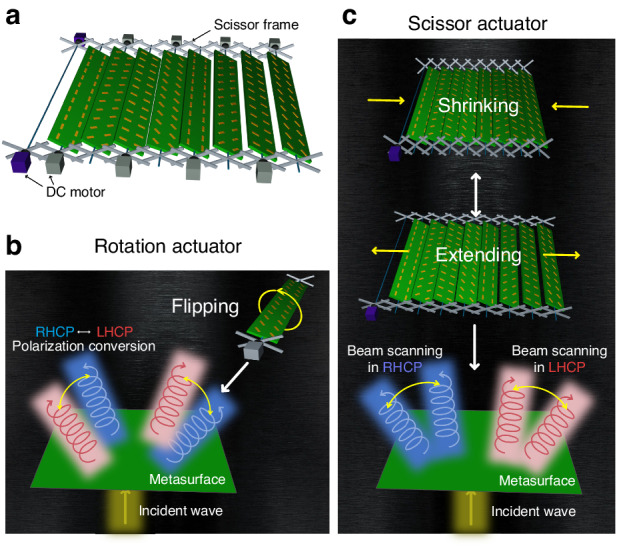
Schematic of the reconfigurable transmissive metasurface with a combination of scissor and rotation actuators for independent control of beam scanning and polarization conversion. **a** The metasurface model. **b** Unit cell rotation controlled for the polarization conversion function. **c** Space controlled by blue DC motors for beam scanning functions

To overcome the challenges associated with designing a metasurface with polarization conversion and beam scanning functionalities, a reconfigurable transmissive metasurface with rotation and scissor actuators that can independently switch the polarization state of an incident wave and steer its propagation direction has been proposed, as shown in Fig. 1a. Such a metasurface is composed of four different types of unit cells (UCs) that can be rotated using the rotation actuator to generate two different phase distributions for polarization conversion, as shown in Fig. 1b. As shown in Fig. 1c, the metasurface can be linearly shrunk or extended using a scissor actuator for beam scanning. This proposed design can overcome the challenges of integrating polarization conversion and beam scanning functionalities on a single platform by using independent actuators to control each function. This method is more practical and efficient than other methods for designing and fabricating reconfigurable metasurfaces with multiple functionalities. In RF applications, the adjustable size of the metasurface renders it adaptable to various environments, including satellite applications^50^. However, in comparison to electrical control, the present version of the proposed metasurface exhibits slower tuning speeds. Consequently, there are applications where swift tuning is not a necessity, such as biosensing^51,52^, wireless power transmission^53,54^, and indoor communication devices^55^.

## Results

### Analytical results

Space-modulated beam scanning using a nonplanar UC geometry widens the beam scanning range using the same design and manufacturing cost. Thus, a space-modulated metasurface provides a wide beam scanning range, which is comparable to that achieved using the phase-modulation mechanism. The space between UCs and the UC excitation coefficients of amplitude and phase can analytically steer a manipulated beam to a certain direction and can be expressed by the following array factor (AF) formula^56^:

1 $${AF}(\theta )=\mathop{\sum }\limits_{n=1}^{N}{e}^{j\frac{2\pi }{{\lambda }_{0}}\left(n-1\right)d\sin \left(\theta \right)+j{\varphi }_{n}}$$

where *d* is the space between UCs and *φ* is the phase.

**Fig. 2:**
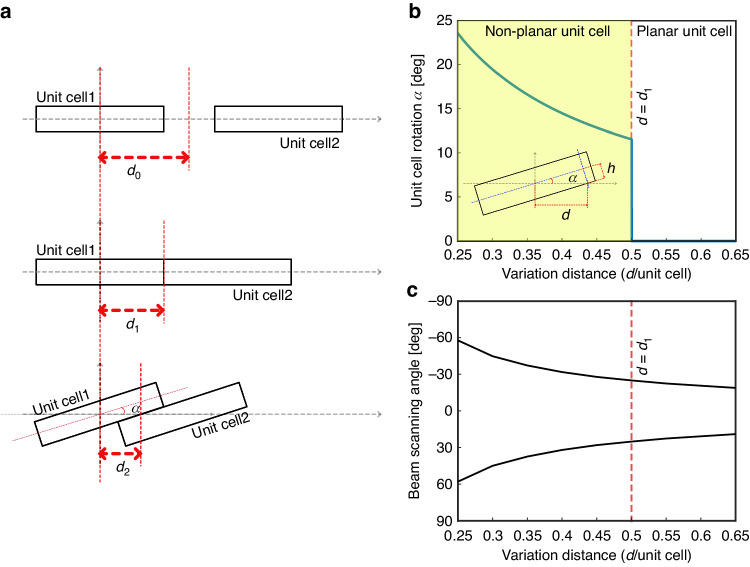
Beam scanning analysis. **a** Geometrical structure representing various states of distance variations. **b** Mathematical relation between UC rotation *α* and the corresponding distance variation *d*. **c** Analytical results beam scanning behavior within distance variation range [*d*_2_, *d*_0_] = [0.25λ_0_, 0.65λ_0_]

Figure 2a shows the conventional planar and nonplanar UC composed of a metasurface, which uses space modulation to steer the beam direction. We assume that the size of the UC is equal to the wavelength *λ*_0_ = 28.5 mm for 10.5 GHz operation frequency and that the distance variation (*d*) is varied within the range [*d*_1_, *d*_0_]. To increase the beam scanning range using the space modulation technique, we combine the UC with a nonplanar geometry with a distant range of *d* = [*d*_2_, *d*_1_], implying that the distance variation range becomes wide. Figure 2b shows the relationship between the UC rotation angle (*α*) and the distance variation (*d*) with the UC size (Fig. S1). By considering the transmission loss of the UC to be higher than −3 dB and the simulation result of the UC, we can analyze the beam scanning range with α = [0, 23.2°] and a distance variation range of *d* = [*d*_2_, *d*_0_] = [0.25λ_0_, 0.65λ_0_]. To predict the beam scanning angle range for a nonplanar UC, we separate the overlay area between the UCs to form a virtual layer. Therefore, the beam angle is predicted as the sum of the AF(*θ*) of both layers. The calculation and analysis details are provided in supplementary note 1. The estimated beam scanning range is from 18° to 60°, as shown in Fig. 2c.

### Numerical results

**Fig. 3:**
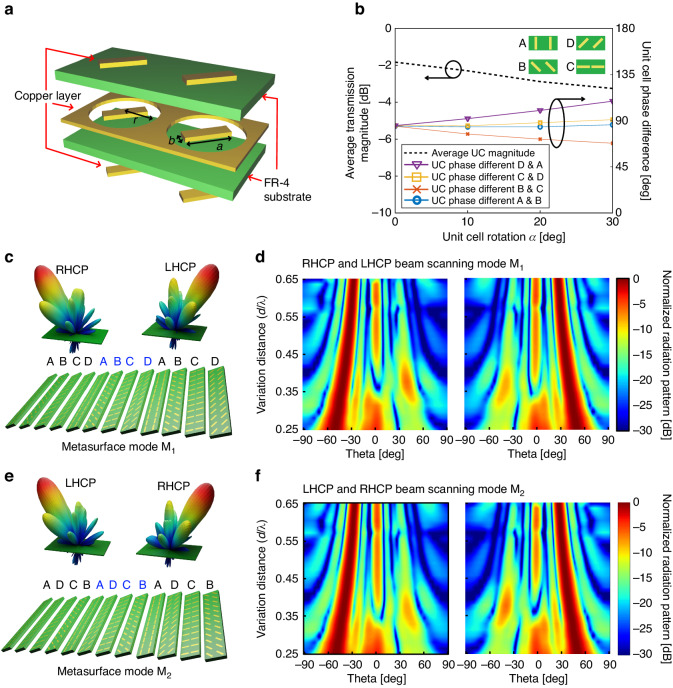
Simulation model and result. **a** 3D UC model. **b** Simulated magnitude and phase of the UC. **c**, **d** Simulated metasurface beam scanning of RHCP and LHCP for polarization mode M_1_. **e**, **f** Simulated metasurface beam scanning of RHCP and LHCP for polarization mode M_2_

Because the beam scanning function is defined based on the space between UCs, we can integrate the polarization conversion function by optimizing the UC phase properties. Conventionally, circular polarization is achieved by combining two orthogonal linearly polarized waves with equal amplitudes and a phase difference of 90°. Specifically, the difference between the phase distributions of the UCs along the *x-* and *y*-axes of a linearly polarized light can create a circularly polarized light by forming a helical structure at a certain phase delay, which is defined by an RHCP and LHCP for clockwise and counterclockwise rotations, respectively, due to the positive and negative phase distributions^57^. Figure 3a shows the design of a two-bit transmission UC (A, B, C and D) with three layers of an I-shaped frequency selective surface and a flame retardant (FR4) substrate with different extinction coefficients. An EM wave can be transmitted through this surface (design parameters: *r* = 3.8 mm, *a* = 6.4 mm, and *b* = 1.4 mm) with an average power ratio of >−2 dB at an operational frequency of 10.5 GHz. The bandwidth ranges from 10.15 to 10.65 GHz (as shown in Fig. S3b, which displays the simulated transmission magnitude of each unit cell). The bandwidth can be enhanced in the Pancharatnam-Berry metasurface by adjusting the number of layers in the design^58^.

The simulated UC structure was optimized using ANSYS Electronic High Frequency Simulation Software (HFSS) with master-slave boundaries and a Floquet port excitation without considering the UC overlay condition (Fig. S2a, b shows the UC and far-field simulation setup with the PML boundary). More detail for the simulation setup is given in supplementary note 2. Figure 3b shows the average transmission loss and phase shift up to −3.3 dB and 20° at a UC rotation angle of α = 30°. We observed the cause of attenuation through the transmission coefficient characteristic of UC A in supplementary note 3. Figure S3c shows that the transmission magnitude decreases from −1.1 dB to −1.8 dB when the loss tangent (tanδ) is increased from 0.005 to 0.020. For the lossless substrate (tanδ = 0), the transmission coefficient is −0.9 dB at 10.5 GHz. Because the proposed prototype is fabricated on the FR4 substrate (tanδ = 0.02), it is expected to be −1.8 dB at 10.5 GHz. For higher transmission efficiency, low loss materials can be used while incurring high costs. Figure S3b shows that the transmission coefficient at 10.5 GHz is decreased from −1.8 dB to −4.1 dB when the incidence angle is increased from 0° to 25°. Because the reflection coefficients under normal and oblique incidences are different, it is obvious that the transmission coefficient decreases with wider incidence angles. In general, the UC is designed under normal circumstances, and the angle-insensitive metasurface can be designed using optimization^59^.

Based on the UC simulation result, we numerically analyzed the designed metasurface with three periods of UCs and compared the resulting beam scanning angle with that obtained analytically for a distance variation of [0.25λ, 0.65λ]. Figure 3c–f displays the far-field simulation results of the metasurface derived for a linearly polarized plane-wave excitation with perfectly matched layer (PML) boundary conditions at an operational frequency of 10.5 GHz. In our simulation setup, we define the UC rotation angle as α_s_ = α + k, where k represents an additional UC rotation. This optimized value of *k*, set as *k* = 1°, is introduced to mitigate conductor shorting issues that may occur due to the assumption of contact between adjacent UC patterns. This assumption was made to simplify the derivation of the relation involving α, particularly when the UC is rotated within the distance range of [0.25λ, 0.50λ]. Fig. S3e, f shows the numerical result for the prediction of the phase distribution of the overlapping area between different UCs.

To validate the performance of the metasurface, we separately simulated the independent reconfigurations of the polarization conversion and beam scanning functions. For an initial metasurface state with a UC array (A-B-C-D-A-B-C-D-A-B-C-D), we consider a polarization mode M_1_; in this case, the phase distribution is simulated to generate RHCP and LHCP at the left and right sides, respectively, in the far-field elevation plane at a fixed distance of *d* = 0.50λ, as shown in Fig. 3c. For the distance variation range [0.25λ, 0.65λ] and a UC rotation angle of α < 30° (shown in Fig. 2b), the RHCP and LHCP beams are steered in ranges from -50° to -22° and from +50° to + 22°, respectively, as shown in Fig. 3d. Figure 3e shows mode M_2_ of the metasurface, wherein the reconfigured UC array is flipped to the state (A-D-C-B-A-D-C-B-A-D-C-B). As a result, at the same distance *d* = 0.50λ, the polarization state is switched from left to right for RHCP and vice versa for LHCP in the elevation plane. Subsequently, the simulated beam scanning ranges for mode M_2_ are from +50° to +22° for RHCP and from −50° to −22° for LHCP, as shown in Fig. 3f. According to the simulation result, the beam scanning range is 28°, which is 14° different from the calculated range because the UC phase shifts under rotation conditions.

### Experimental Results

**Fig. 4:**
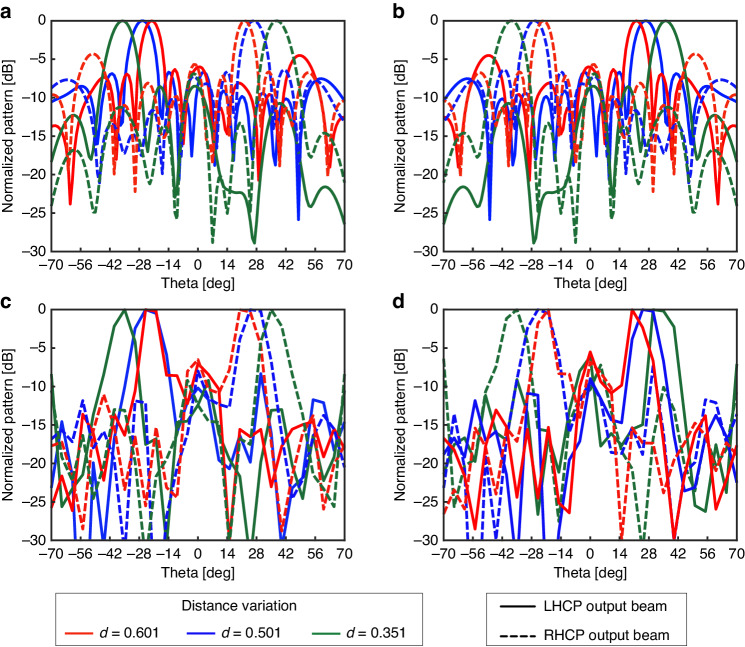
RHCP and LHCP output beam results. **a**, **b** Simulation results for modes M_1_ and M_2_. **c**, **d** Measurement results for modes M_1_ and M_2_

To verify the metasurface performance, we fabricated a 12 × 12 array of unit cells (UCs) using a printed circuit board. We conducted measurements of the radiation pattern at a specific reconfiguration state of the UCs, considering three distances (spacing) between them: d = 0.35λ, 0.50λ, and 0.60λ. The measurements were carried out for both polarization modes, M1 and M2. We anticipated that the observed radiation pattern would align with the simulation results, as shown in Fig. 4a, b. Two horn antennas were used to observe the EM wave transmitted through the metasurface. A transmissive horn antenna generates a linearly polarized plane-wave source, which radiates from the backside of the metasurface. Another horn antenna is used to measure the magnitude and phase with horizontal and vertical polarization to observe the converted circularly polarized wave at the front side of the metasurface and derive the radiation pattern, as shown in Fig. 4c, d. Supplementary note 4 and Fig. S4 show the details of the measurement setup. The properties of the EM wave propagating from the transmitter to the receiver antenna were recorded by an Anritsu MS2038C vector network analyzer and plotted as a radiation pattern with a normalized magnitude and 5° scanning resolution.

As a result, the measured and simulated RHCP and LHCP beam scanning angles (θ) of the metasurface at modes M_1_ and M_2_ are similar (37° and 22°, respectively) with UC spacings of 0.35λ and 0.60λ, respectively. In contrast, at *d* = 0.50λ, the measured beam angle is slightly shifted (by 1°) from the simulated beam angle (θ = 27°) for the RHCP wave at mode M_1_ due to the tolerance of the measurement setup at *d* = 0.35λ. Evidently, the side-loop magnitudes at the RHCP and LHCP states are similar (~ −10 dB); this result confirms the polarization conversion performance of the fabricated metasurface.

## Discussion

A reconfigurable transmissive metasurface using new scissor and rotation actuators with DC motor and scissor mechanism integration is proposed, and the polarization switching (between RHCP and LHCP beams) performance of the metasurface for a forward linearly polarized incident wave is both numerically and experimentally confirmed. This proposed metasurface can perform controlled beam scanning at multiple angles. The metasurface is designed to independently control the space between UCs and their phase with displacement and rotation. The beam scanning performance is significantly improved by the spacing modulation technique implemented using a metasurface with a nonplanar UC and an overlaid phase delay. Furthermore, the scanning performance of the space-modulated metasurface is comparable to that achieved using the phase-modulation mechanism.

## Materials and methods

**Fig. 5:**
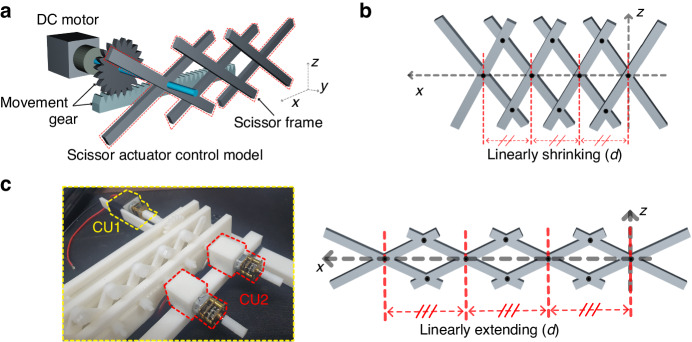
Scissor actuator: conception and design. **a**, **b** Scissor actuator design model and conception. **c** Mechanism of the independent control function

To explore the design tuning mechanism for transmissive metasurfaces, we developed a mechanical tuner using a scissor mechanism, as shown in Fig. 5a. This mechanical structure consists of two folding supports or sticks arranged in a crisscross ‘×‘ pattern. In Fig. 5b, it can be observed that a movement on the first cross results in linear movement of the adjacent crosses due to the transformative nature of the scissor mechanism.

By attaching each unit cell (UC) to the center of each cross, we can control the spacing between the UCs using shrinking and extending mechanisms incorporated into the scissor frame. The frame reconfiguration is controlled by a DC motor acting as control unit 1 (CU 1), which pulls and pushes at the edge cross of the frame based on the scissor mechanism concept. Let *x* be the reconfiguration by CU1. The tuning spacing *d* can be calculated as (x/n), where *n* is the amount of cross demonstrated in the frame design. Furthermore, the scissor frame can be conveniently installed in a side area of the metasurface, allowing for compatibility with the metasurface design for transmission functions. To achieve rotation, we employed an array of rotation control units, referred to as CU 2, positioned between the two aligned scissor frames and the metasurface. These rotation control units enable the UCs to be independently rotated in relation to CU 2. Figure 5c shows the fabricated rotation and scissor actuators. The scissor frame was fabricated using a polylactic acid filament, which was printed in pieces using a 3DWOX 7X 3D printer and then assembled using plastic screws. The experiment and some design properties are described in supplementary note 5. In addition, we determined the tuning speed of the metasurface with the fabricated scissor frame (the control schematic and experimental environment are shown in Fig. S5), which was produced by 3D printing. The distance between 12 UCs could be tuned at a speed of up to 0.16λ/s using a 5 V DC control signal^59^.

### Supplementary information

Supplementary Information
