# Supplementary material for: Reconfigurable transmissive metasurface with a combination of scissor and rotation actuators for independently controlling beam scanning and polarization conversion
Source: Microsyst Nanoeng. 2024 Mar 21;10:40. doi: 10.1038/s41378-024-00671-y (PMC10958044; doi:10.1038/s41378-024-00671-y)
Supplement: Supplementary file 1 — Supplementary Information [file 41378_2024_671_MOESM1_ESM.docx]

**Supporting Information**

1. UC Rotation and Beam Scanning

Unlike a planar UC, a nonplanar UC has an element steering factor, which can increase the amplitude of the scattered side beam. However, the use of a nonplanar UC also has a trade-off in power due to the oblique incidence angle caused by the unnormalized UC with respect to the incident wave direction. Hence, in this section, we present the analysis of a rotatable UC for obtaining the best result. Figure S1a shows the geometrical structure of the rotating UC, wherein the distance varies with a minimal value of the rotation angle α. Considering the rotation angle effect, we optimized the distance variation by combining both planar and nonplanar UCs. The rotation angle is defined as:

|  | $\left\{ \begin{aligned} \alpha= \sin^{-1} \frac{h}{d} for 0<d<d_{1} \\ \\ \alpha=0 \mathrm{for} d>d_{1} \\ \end{aligned} \right.$ |  | Eq. S1 |
| --- | --- | --- | --- |

For a conventional planar spaced-modulated metasurface, the beam direction can be predicted using the AF equation. Conversely, for a spaced-modulated metasurface with nonplanar UCs, overlay areas exist between the UCs at 0 < *d* < *d*_1_. Figure S1b shows the analyzed overlaid and nonoverlaid distances (d’ and d”, respectively), which can be described as follows:

For a UC length and width defined as UC and 2h, respectively, and given values of *d* and *α*, we obtain

$2d = d’+d”$,$x_{1}+x_{2}=UC$

We obtain the following from the blue triangle:

|  | $d"$ | = | $2 x_{2} cos(\alpha)$ |  | (1) |
| --- | --- | --- | --- | --- | --- |

We obtain the following from the red triangle:

|  | $x_{1}$ | = | $\sqrt{d^{2}-h^{2}}$ |  |  |
| --- | --- | --- | --- | --- | --- |
| $\Rightarrow$ | $x_{2}$ | = | $UC-x_{1}$ =$UC-\sqrt{d^{2}-h^{2}}$ |  |  |

By replacing $x_{2}$ in (1), we obtain:

|  | $d"$ | = | $2(UC-\sqrt{d^{2}-h^{2}}) cos(\alpha)$ |  |  |
| --- | --- | --- | --- | --- | --- |
| and | $d'$ | = | $2d-d" = 2d-2(UC-\sqrt{d^{2}-h^{2}}) cos(\alpha)$ |  |  |

We assume no coupling between the UCs and divide the phase distribution into two planar phase layers 1 and 2 based on the defined overlaid and nonoverlaid areas with distances d’ and d” (Fig. S1c-d). To predict the nonplanar space modulation of the UCs, we separately calculate the AF of each phase layer (AF_1_ and AF_2_) in the radiation pattern and add the resulting values to determine the beam direction:

|  | ${AF}_{planar}$ | = | $AF(\theta,d,\varphi)$ |  |  |
| --- | --- | --- | --- | --- | --- |
|  | ${AF}_{non-planar}$ | = | ${AF}_{1}\left( \theta,d^{'},d",\varphi\right)+{AF}_{2}\left( \theta,d^{'},d",\varphi\right)$ |  |  |

**
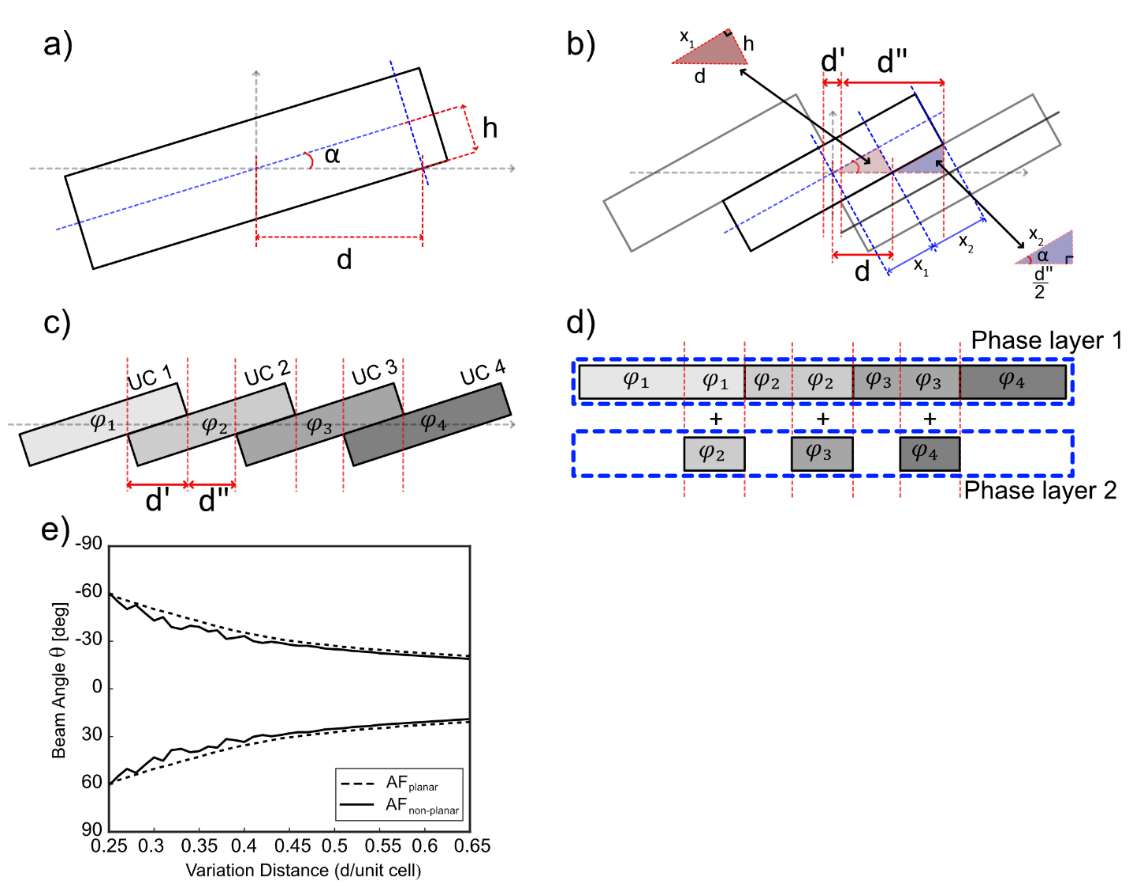
** Figure S1e shows the calculated beam angle of a conventional two-bit planar metasurface and that of the proposed nonplanar metasurface. The nonplanar UC still performs beam scanning from 20° to 60° with a slight vibration due to the same phase distribution of phase layers 1 and 2.

Fig. S1 a-b Geometrical analysis of the UC. c-e Beam direction angle analysis and results.

1. Simulation Setup


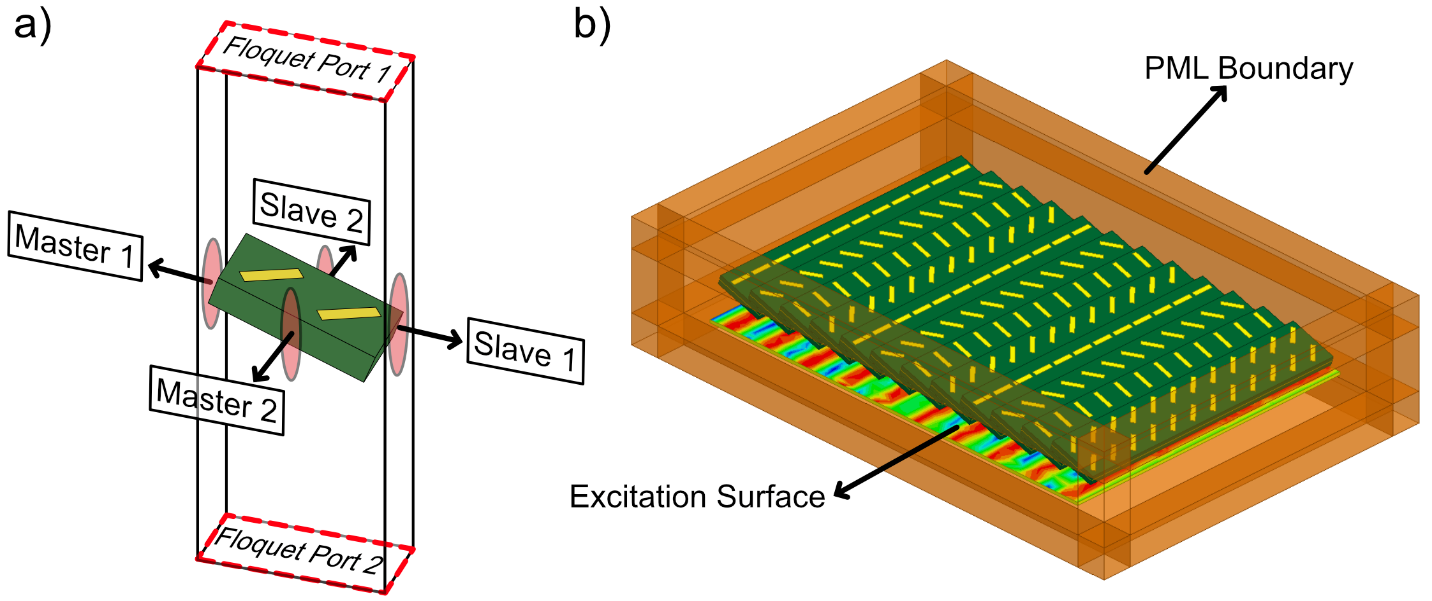


Fig. S2 a-b UC and full structure simulation setup.

To demonstrate the concept of the proposed metasurface, we present the simulation results for both the UC and metasurface in Ansys HFSS simulation software.

- UC simulation

For the UC simulation, the 3D model of the polarization conversion unit cell is simulated under two pairs of master-slave boundary conditions, as shown in Fig. S2a. In addition, two Floquet ports are assigned for two-port S-parameter network analysis with two orthogonal electric fields for circular polarization observation. Finally, we simulate the UC in different rotation states and export the result of the transmission polarization conversion magnitude, as shown in Fig. 2b.

- Full structure simulation

For the full structure simulation, the metasurface with a finite number of unit cells is simulated with perfect matching layer (PML) boundary conditions, as shown in Fig. S2b. To characterize the RHCP and LHCP radiation patterns, an incident plane wave is assigned on an excitation surface to generate an LP plane wave toward the metasurface. Using the setup for the full structure simulation, we can obtain the beam scanning result, as shown in Fig. 2e-f.

1. Unit Cell Analysis


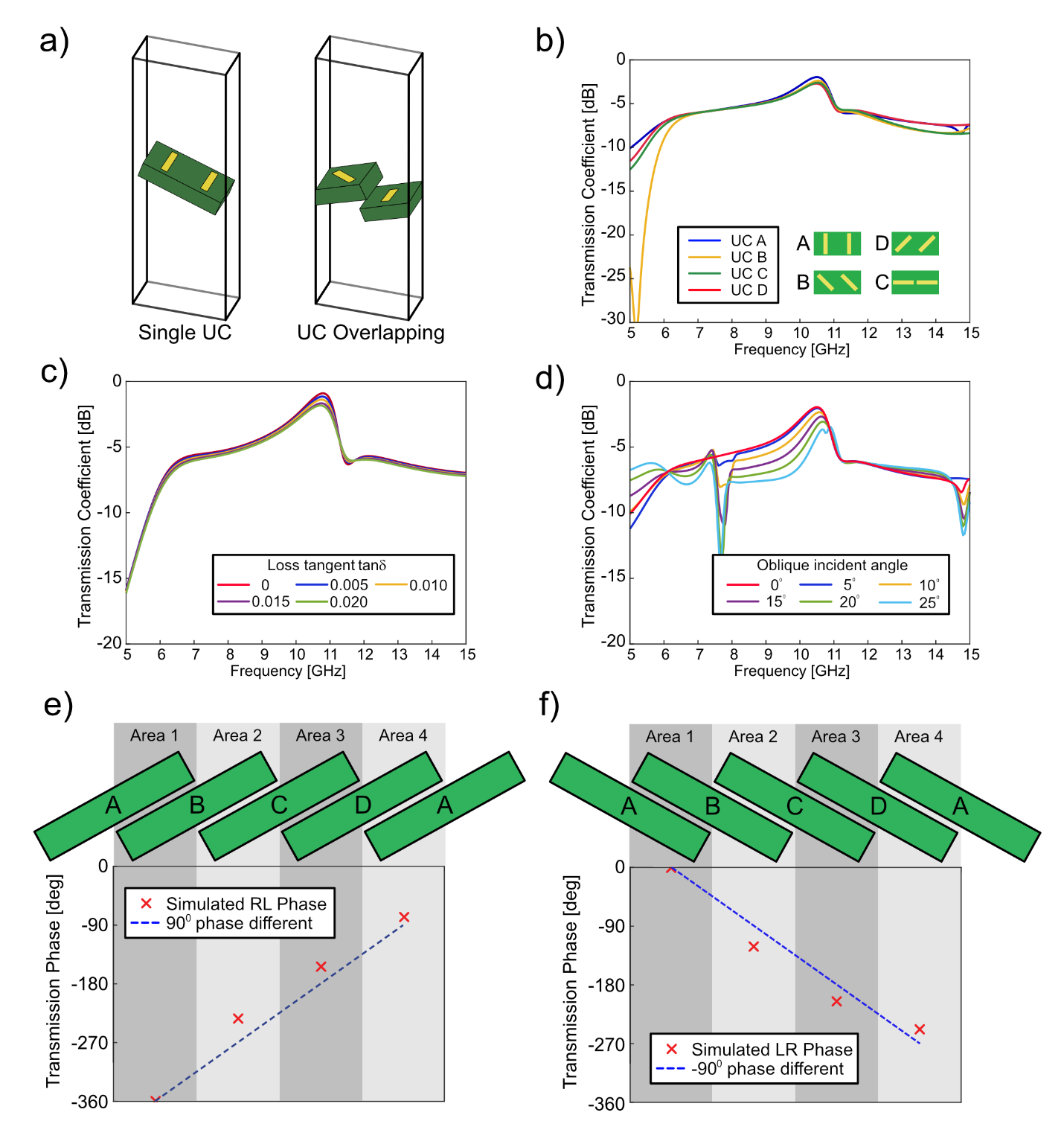


Fig. S3 UC simulation: a Simulation model. b Transmission magnitude of each UC. c-d Transmission magnitudes at different loss tangent and oblique incident angles. e-f Transmission phases of the overlap between UCs.

Conventional electromagnetic simulation utilizes a single UC with an infinite boundary to represent the periodicity of the structure under investigation. However, this assumption can be limited by the complex structure and different UC types of scenario structures. The proposed metasurface is constructed with two states of planar and nonplanar UCs, and UC overlap occurs when the spacing is smaller than half the size of the UC. Figure S3a shows the simulation model for both a single UC and overlapping UCs for the prediction of phase distribution using periodic boundaries. The setup environment is depicted in Fig. S2a.

- Single UC analysis

Figure S3b shows the simulated transmission magnitudes of the optimized 2-bit unit cell (UC) under planar conditions operating at a frequency of 10.5 GHz. Under normal incident conditions with a UC rotation angle of 0°, all UCs exhibit transmissions ranging from -1.41 to -2.05 dB, averaging -1.67 dB for the lossy FR-4 substrate with tanδ = 0.02. Additionally, the bandwidth is limited to 0.5 GHz due to the phase pattern of UC B. In Fig. S3b, we conducted simulations of the transmission magnitudes for UC A under a low loss FR-4 condition with a variation in tanδ from 0 to 0.02. The results show transmission magnitudes of -0.9, -1.15, -1.38, -1.66 and -1.8 dB for tanδ values of 0, 0.005, 0.010, 0.015 and 0.02, respectively. This further underscores the influence of the loss tangent of the FR-4 substrate on the observed transmission characteristics. Because the proposed prototype is constructed on the FR4 substrate (tanδ =0.02), its transmission is expected to be -1.8 dB at 10.5 GHz. Figure S3c depicts the transmission characteristics of UC A as the oblique incident rate increases. As a result, increasing the oblique incident angle from 0° to 25° can decrease the transmission from -1.8 to -4.1 dB. As mentioned in the manuscript, the efficiency of the metasurface can be improved through the optimization of the design.

- UC overlapping analysis

It is difficult to accurately predict the magnitude of electromagnetic responses within nonplanar, overlapping geometries due to the intricate interference patterns and complex interactions that arise in these regions. However, when focusing on distinct electromagnetic phenomena, we conducted simulations to predict the characteristics of the phase differences in overlapping areas. Without considering the coupling and oblique incident effect, we assume that the overlap between UC stacks the UC into multiple layers. Figure S3e-f shows the simulated transmission phases in overlapping areas 1-4 for propagation directions RL and LR at the smallest distance variation of d = 0.25λ_0_. The results indicate that the phase difference in each area remains consistent at approximately 90°.

1. Environment for Measuring Circular Polarization


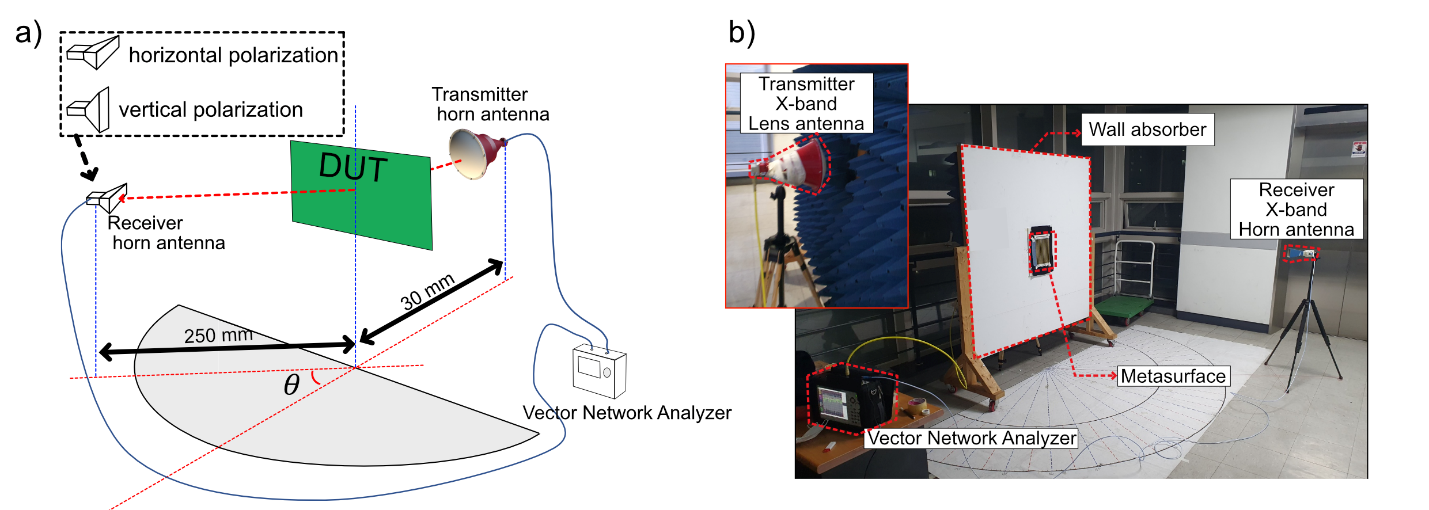


Fig. S4 a-b Conception and practical setup of the circular polarization measurement.

As mentioned in the paper, circular polarization can be induced by forming a helical structure with a certain phase delay from the ***x-*** and *y*-axes of linear polarization. We observed the radiation pattern of the RHCP and LHCP beams by combining the phases and magnitudes of the horizontally and vertically polarized signals of the receiver horn antenna. Figure S4a shows the conceptual measurement setup of the metasurface. An X-band transmitter lens antenna is placed at the backside 30 mm behind the metasurface to generate a linearly polarized plane-wave that propagates to the backside of the metasurface. Another horn antenna is placed 250 mm at the front of the metasurface to observe the characteristics of the EM wave manipulated by the metasurface in different polarization states (i.e., horizontal and vertical polarization states). Figure S4b shows the practical measurement setup, which includes an additional wall absorber to mitigate interference from the environment.

1. Scissor Actuator Design


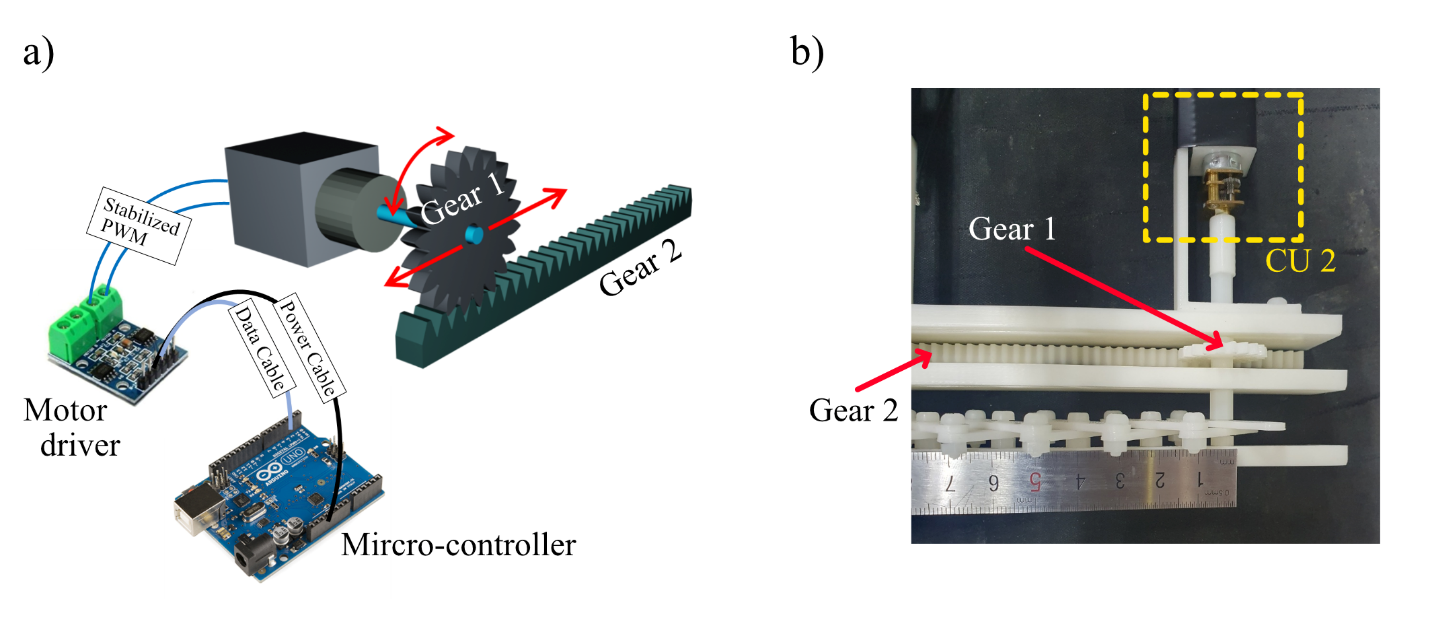


Fig. S5: Scissor actuator. a Control schematic. b Control experiment

To effectively control the shrinking and extension of the scissor actuator, we utilized a micro-DC motor with mechanical parameters adjusted via a microcontroller device. Figure S5a shows the schematic of the scissor actuator with a rack gear system comprising gear 1 and gear 2; gear 1 has 20 teeth and a pitch diameter of *d*_g_ = 20 mm. For electrical control of the DC motor, we used the Microcontroller Arduino Uno ATMEGA328P-PU, along with a motor driver L9110B, to stabilize a 5 V DC electrical signal with vibration elimination. This signal was used to control the rotation of a GA12-N20 micromotor for pulling a 12 crisscrossed scissor frame, as shown in Fig. S5b. As a result, the rotation speeds could be precisely controlled, and we achieved a tuning speed of 60 mm/s. Thus, the distance between the 12 crisscrossed scissor UCs was controlled to *d* = 5 mm/s.
